# Supplementary material for: Topical fluoride hesitancy and opposition are significantly and positively associated: A cross-sectional study
Source: PLoS One. 2025 Apr 30;20(4):e0322027. doi: 10.1371/journal.pone.0322027 (PMC12043125; doi:10.1371/journal.pone.0322027)
Supplement: S1 Table — (DOCX) [file pone.0322027.s001.docx]

**S1 Table.** Description of Sociodemographic Characteristics of Surveyed Caregivers and Domain-Specific Hesitancy with P-Values Indicating Differences in Sociodemographic Characteristics by Domain-Specific Topical Fluoride Hesitancy Category (N=1,042)

| Sociodemographic Characteristic | Necessity  Mean±SD or N (%) | | | *p*-Value | Chemicals  Mean±SD or N (%) | | | *p*-Value | Harm  Mean±SD or N (%) | | | *p*-Value | Uncertainty  Mean±SD or N (%) | | | *p*-Value | Distrust  Mean±SD or N (%) | | | *p*-Value |
| --- | --- | --- | --- | --- | --- | --- | --- | --- | --- | --- | --- | --- | --- | --- | --- | --- | --- | --- | --- | --- |
|  | **<1 n=195 18.8%** | **1-2 n=787 75.7%** | **>2 n=58 5.6%** |  | **<1 n=698 68.7%** | **1-2 n=248 24.4%** | **>2 n=70 6.9%** |  | **<1 n=815 80.5%** | **1-2 n=144 14.2%** | **>2 n=54 5.3%** |  | **<1 n=705 69.9%** | **1-2 n=251 24.9%** | **>2 n=52 5.2%** |  | **<1 n=758 74.8%** | **1-2 n=224 22.1%** | **>2 n=32 3.2%** |  |
| Child Gender |  |  |  | .30 |  |  |  | .66 |  |  |  | .23 |  |  |  | .76 |  |  |  | .27 |
| Boy | 99 (51.3) | 384 (49.2) | 34 (59.6) |  | 338 (48.9) | 124 (50.6) | 38 (54.3) |  | 404 (50.1) | 63 (44.4) | 31 (57.4) |  | 340 (48.8) | 128 (51.4) | 25 (48.1) |  | 373 (49.7) | 105 (47.3) | 20 (62.5) |  |
| Girl | 94 (48.7) | 396 (50.8) | 23 (40.4) |  | 353 (51.1) | 121 (49.4) | 32 (45.7) |  | 403 (49.9) | 79 (55.6) | 23 (42.6) |  | 357 (51.2) | 121 (48.6) | 27 (51.9) |  | 377 (50.3) | 117 (52.7) | 12 (37.5) |  |
| Child Age, *mean±SD* | 7.7±4.6 | 7.8±4.3 | 6.6±4.3 | .88 | 7.9±4.3 | 7.2±4.4 | 7.2±4.6 | .41 | 7.9±4.4 | 7.4±4.3 | 5.7±3.6 | .21 | 7.9±4.4 | 7.3±4.4 | 6.7±3.7 | .58 | 7.9±4.3 | 7.4±4.4 | 5.8±4.1 | .20 |
| Child Health Insurance Type |  |  |  | **.046*** |  |  |  | **.02*** |  |  |  | **.047*** |  |  |  | **.002**** |  |  |  | **.008**** |
| Private | 90 (46.9) | 279 (36.0) | 19 (33.3) |  | 287 (41.8) | 72 (29.1) | 22 (32.4) |  | 328 (40.7) | 37 (26.4) | 16 (30.2) |  | 295 (42.3) | 73 (29.7) | 11 (21.6) |  | 305 (40.8) | 67 (30.3) | 9 (29.0) |  |
| Medicaid/public | 96 (50.0) | 441 (57.0) | 32 (56.1) |  | 361 (52.5) | 155 (62.8) | 39 (57.4) |  | 430 (53.3) | 90 (64.3) | 33 (62.3) |  | 361 (51.8) | 154 (62.6) | 35 (68.6) |  | 397 (53.1) | 140 (63.3) | 16 (51.6) |  |
| No insurance | 2 (1.0) | 19 (2.5) | 1 (1.8) |  | 12 (1.7) | 7 (2.8) | 3 (4.4) |  | 17 (2.1) | 4 (2.9) | 1 (1.9) |  | 13 (1.9) | 8 (3.3) | 1 (2.0) |  | 14 (1.9) | 6 (2.7) | 2 (6.5) |  |
| Other | 4 (2.1) | 35 (4.5) | 5 (8.8) |  | 27 (3.9) | 13 (5.3) | 4 (5.9) |  | 31 (3.8) | 9 (6.4) | 3 (5.7) |  | 28 (4.0) | 11 (4.5) | 4 (7.8) |  | 32 (4.3) | 8 (3.6) | 4 (12.9) |  |
| Caregiver Gender |  |  |  | **<.001***** |  |  |  | **.02*** |  |  |  | .07 |  |  |  | .19 |  |  |  | .31 |
| Man | 31 (16.8) | 155 (21.2) | 14 (28.6) |  | 135 (20.1) | 51 (22.4) | 14 (21.5) |  | 152 (19.5) | 34 (26.4) | 13 (25.5) |  | 134 (19.7) | 56 (24.1) | 9 (18.8) |  | 144 (19.9) | 155 (74.2) | 5 (17.2) |  |
| Woman | 152 (82.2) | 573 (78.4) | 34 (69.4) |  | 532 (79.4) | 175 (76.8) | 50 (76.9) |  | 623 (79.9) | 94 (72.9) | 38 (74.5) |  | 541 (79.7) | 175 (75.4) | 38 (79.2) |  | 577 (79.7) | 51 (24.4) | 24 (82.8) |  |
| Non-binary or third gender | 2 (1.1) | 3 (0.4) | 0 (0.0) |  | 3 (0.4) | 2 (0.9) | 0 (0.0) |  | 5 (0.6) | 0 (0.0) | 0 (0.0) |  | 4 (0.6) | 0 (0.0) | 1 (2.1) |  | 3 (0.4) | 2 (1.0) | 0 (0.0) |  |
| Prefer to self-identify as other | 0 (0.0) | 0 (0.0) | 1 (2.0) |  | 0 (0.0) | 0 (0.0) | 1 (1.5) |  | 0 (0.0) | 1 (0.8) | 0 (0.0) |  | 0 (0.0) | 1 (0.4) | 0 (0.0) |  | 0 (0.0) | 1 (0.5) | 0 (0.0) |  |
| Caregiver Age, *mean±SD* | 42.9±7.9 | 41.8±8.4 | 42.4±9.2 | .48 | 42.0±8.1 | 41.7±8.9 | 43.5±8.9 | .09 | 42.0±8.2 | 42.2±9.8 | 41.8±7.2 | .40 | 42.3±8.1 | 41.5±9.2 | 41.4±7.2 | .57 | 42.1±8.2 | 41.8±8.6 | 42.4±9.0 | .66 |
| Caregiver Race |  |  |  | **<.001***** |  |  |  | **<.001***** |  |  |  | **<.001***** |  |  |  | **<.001***** |  |  |  | **<.001***** |
| White | 137 (70.3) | 382 (48.5) | 22 (37.9) |  | 408 (58.5) | 96 (38.7) | 38 (54.3) |  | 469 (62.1) | 51 (41.8) | 21 (42.0) |  | 411 (62.5) | 108 (48.6) | 21 (45.7) |  | 434 (61.6) | 93 (47.2) | 15 (55.6) |  |
| Black | 5 (2.6) | 65 (8.3) | 9 (15.5) |  | 48 (6.9) | 26 (10.5) | 4 (5.7) |  | 55 (7.3) | 15 (12.3) | 8 (16.0) |  | 46 (7.0) | 26 (11.7) | 6 (13.0) |  | 54 (7.7) | 23 (11.7) | 1 (3.7) |  |
| Asian | 19 (9.7) | 139 (17.7) | 9 (15.5) |  | 92 (13.2) | 59 (23.8) | 15 (21.4) |  | 112 (14.8) | 37 (30.3) | 16 (32.0) |  | 97 (14.7) | 53 (23.9) | 15 (32.6) |  | 106 (15.0) | 50 (25.4) | 10 (37.0) |  |
| Multiple/Other | 19 (9.7) | 118 (15.0) | 6 (10.3) |  | 101 (14.5) | 36 (14.5) | 6 (8.6) |  | 119 (15.8) | 19 (15.6) | 5 (10.0) |  | 104 (15.8) | 35 (15.8) | 4 (8.7) |  | 111 (15.7) | 31 (15.7) | 1 (3.7) |  |
| Caregiver Ethnicity |  |  |  | .69 |  |  |  | .36 |  |  |  | .90 |  |  |  | .88 |  |  |  | .99 |
| Hispanic | 23 (12.6) | 101 (14.0) | 5 (10.2) |  | 93 (14.0) | 30 (13.5) | 5 (7.7) |  | 101 (13.2) | 19 (14.6) | 7 (13.7) |  | 88 (13.2) | 33 (14.3) | 6 (12.5) |  | 95 (13.3) | 28 (13.5) | 4 (13.8) |  |
| Non-Hispanic | 160 (87.4) | 620 (86.0) | 44 (89.8) |  | 571 (86.0) | 192 (86.5) | 60 (92.3) |  | 666 (86.8) | 111 (85.4) | 44 (86.3) |  | 581 (86.8) | 197 (85.7) | 42 (87.5) |  | 619 (86.7) | 179 (86.5) | 25 (86.2) |  |
| Caregiver Education |  |  |  | **<.001***** |  |  |  | **.02*** |  |  |  | **<.001***** |  |  |  | **<.001***** |  |  |  | **.02*** |
| Less than high school diploma | 1 (0.5) | 20 (2.7) | 5 (10.4) |  | 13 (1.9) | 8 (3.5) | 4 (6.3) |  | 16 (2.1) | 4 (3.1) | 5 (10.0) |  | 15 (2.2) | 7 (3.0) | 3 (6.3) |  | 16 (2.2) | 6 (2.9) | 3 (10.3) |  |
| High school diploma or equivalent | 17 (9.2) | 97 (13.3) | 10 (20.8) |  | 79 (11.8) | 36 (15.9) | 8 (12.5) |  | 87 (11.2) | 26 (20.2) | 9 (18.0) |  | 77 (11.4) | 32 (13.9) | 13 (27.1) |  | 88 (12.2) | 27 (13.1) | 7 (24.1) |  |
| Some college or 2-year college degree | 46 (25.0) | 218 (29.9) | 13 (27.1) |  | 179 (26.7) | 75 (33.2) | 22 (34.4) |  | 214 (27.5) | 43 (33.3) | 19 (38.0) |  | 181 (26.7) | 77 (33.3) | 18 (37.5) |  | 198 (27.3) | 71 (34.5) | 7 (24.1) |  |
| 4-year college degree | 47 (25.5) | 182 (24.9) | 11 (22.9) |  | 170 (25.4) | 54 (23.9) | 16 (25.0) |  | 206 (26.5) | 27 (20.9) | 6 (12.0) |  | 175 (25.8) | 57 (24.7) | 6 (12.5) |  | 186 (25.7) | 50 (24.3) | 4 (13.8) |  |
| More than 4-year college degree | 73 (39.7) | 213 (29.2) | 9 (18.8) |  | 229 (34.2) | 53 (23.5) | 14 (21.9) |  | 255 (32.8) | 29 (22.5) | 11 (22.0) |  | 229 (33.8) | 58 (25.1) | 8 (16.7) |  | 236 (32.6) | 52 (25.2) | 8 (27.6) |  |
| Parenting Style Scale, *mean±SD* | 0.5±0.5 | 0.7±0.5 | 0.7±0.8 | **<.001***** | 0.6±0.5 | 0.7±0.5 | 0.6±0.6 | .06 | 0.6±0.5 | 0.7±0.6 | 0.5±0.5 | .06 | 0.6±0.5 | 0.7±0.5 | 0.6±0.6 | .13 | 0.6±0.5 | 0.7±0.5 | 0.9±0.7 | **<.001***** |
| Caregiver Religiosity |  |  |  | **.02*** |  |  |  | **.001**** |  |  |  | **.002**** |  |  |  | **.009**** |  |  |  | **.02*** |
| Very important | 49 (26.3) | 264 (35.9) | 23 (46.0) |  | 220 (32.5) | 90 (39.0) | 26 (39.4) |  | 250 (31.8) | 58 (43.6) | 26 (50.0) |  | 218 (31.8) | 94 (40.3) | 21 (42.0) |  | 241 (33.0) | 80 (38.3) | 13 (41.9) |  |
| Somewhat important | 40 (21.5) | 190 (25.8) | 11 (22.0) |  | 155 (22.9) | 62 (26.8) | 24 (36.4) |  | 189 (24.1) | 38 (28.6) | 13 (25.0) |  | 161 (23.5) | 68 (29.2) | 11 (22.0) |  | 168 (23.0) | 64 (30.6) | 9 (29.0) |  |
| Not too important | 39 (21.0) | 115 (15.6) | 7 (14.0) |  | 117 (17.3) | 41 (17.7) | 4 (6.1) |  | 139 (17.7) | 16 (12.0) | 7 (13.5) |  | 122 (17.8) | 31 (13.3) | 9 (18.0) |  | 130 (17.8) | 26 (12.4) | 6 (19.4) |  |
| Not at all important | 58 (31.2) | 167 (22.7) | 9 (18.0) |  | 184 (27.2) | 38 (16.5) | 12 (18.2) |  | 207 (26.4) | 21 (15.8) | 6 (11.5) |  | 185 (27.0) | 40 (17.2) | 9 (18.0) |  | 192 (26.3) | 39 (18.7) | 3 (9.7) |  |
| Caregiver Political Ideology |  |  |  | **<.001***** |  |  |  | **<.001***** |  |  |  | **<.001***** |  |  |  | **<.001***** |  |  |  | **<.001***** |
| Very conservative | 4 (2.2) | 18 (2.5) | 10 (20.4) |  | 12 (1.8) | 11 (5.0) | 9 (14.3) |  | 16 (2.1) | 6 (4.7) | 10 (20.8) |  | 13 (1.9) | 13 (5.9) | 6 (12.5) |  | 14 (2.0) | 12 (6.1) | 6 (20.0) |  |
| Conservative | 14 (7.7) | 92 (13.0) | 4 (8.2) |  | 72 (11.0) | 26 (11.7) | 12 (19.0) |  | 82 (10.8) | 16 (12.4) | 10 (20.8) |  | 74 (11.1) | 28 (12.7) | 6 (12.5) |  | 78 (11.0) | 26 (13.1) | 5 (16.7) |  |
| Moderate | 49 (26.8) | 308 (43.4) | 19 (38.8) |  | 257 (39.1) | 95 (42.8) | 24 (38.1) |  | 300 (39.4) | 56 (43.4) | 19 (39.6) |  | 256 (38.3) | 95 (43.0) | 23 (47.9) |  | 279 (39.2) | 86 (43.4) | 10 (33.3) |  |
| Liberal | 62 (33.9) | 184 (26.0) | 7 (14.3) |  | 190 (28.9) | 52 (23.4) | 12 (19.0) |  | 219 (28.7) | 31 (24.0) | 4 (8.3) |  | 195 (29.1) | 55 (24.9) | 4 (8.3) |  | 208 (29.2) | 42 (21.2) | 4 (13.3) |  |
| Very liberal | 54 (29.5) | 107 (15.1) | 9 (18.4) |  | 126 (19.2) | 38 (17.1) | 6 (9.5) |  | 145 (19.0) | 20 (15.5) | 5 (10.4) |  | 131 (19.6) | 30 (13.6) | 9 (18.8) |  | 133 (18.7) | 32 (16.2) | 5 (16.7) |  |
| Annual Household Income |  |  |  | **.02*** |  |  |  | **.002**** |  |  |  | **.001**** |  |  |  | **<.001***** |  |  |  | .14 |
| <$15,000 | 8 (4.4) | 49 (7.0) | 5 (10.4) |  | 38 (5.8) | 19 (8.8) | 5 (8.2) |  | 47 (6.2) | 13 (10.6) | 2 (4.1) |  | 40 (6.1) | 16 (7.2) | 6 (13.3) |  | 46 (6.5) | 13 (6.5) | 3 (11.1) |  |
| $15,000 to <$25,000 | 11 (6.1) | 73 (10.4) | 5 (10.4) |  | 55 (8.4) | 27 (12.5) | 5 (8.2) |  | 66 (8.7) | 16 (13.0) | 5 (10.2) |  | 54 (8.2) | 29 (13.1) | 4 (8.9) |  | 63 (8.9) | 21 (10.6) | 3 (11.1) |  |
| $25,000 to <$50,000 | 36 (19.9) | 151 (21.4) | 14 (29.2) |  | 126 (19.2) | 54 (25.0) | 21 (34.4) |  | 146 (19.3) | 32 (26.0) | 21 (42.9) |  | 122 (18.5) | 60 (27.0) | 17 (37.8) |  | 139 (19.7) | 52 (26.1) | 9 (33.3) |  |
| $50,000 to <$75,000 | 26 (14.4) | 129 (18.3) | 9 (18.8) |  | 112 (17.1) | 43 (19.9) | 8 (13.1) |  | 138 (18.2) | 17 (13.8) | 8 (16.3) |  | 117 (17.7) | 38 (17.1) | 8 (17.8) |  | 120 (17.0) | 37 (18.6) | 6 (22.2) |  |
| $75,000 to <$100,000 | 16 (8.8) | 83 (11.8) | 4 (8.3) |  | 75 (11.5) | 27 (12.5) | 3 (4.9) |  | 92 (12.2) | 9 (7.3) | 4 (8.2) |  | 79 (12.0) | 24 (10.8) | 2 (4.4) |  | 80 (11.4) | 25 (12.6) | 0 (0.0) |  |
| $100,000 to <$150,000 | 34 (18.8) | 112 (15.9) | 6 (12.5) |  | 117 (17.9) | 24 (11.1) | 11 (18.0) |  | 123 (16.2) | 24 (19.5) | 4 (8.2) |  | 112 (16.9) | 35 (15.8) | 3 (6.7) |  | 120 (17.0) | 28 (14.1) | 3 (11.1) |  |
| ≥$150,000 | 50 (27.6) | 107 (15.2) | 5 (10.4) |  | 132 (20.2) | 22 (10.2) | 8 (13.1) |  | 145 (19.2) | 12 (9.8) | 5 (10.2) |  | 137 (20.7) | 20 (9.0) | 5 (11.1) |  | 136 (19.3) | 23 (11.6) | 3 (11.1) |  |

*Note*: Boldface indicates statistical significance (**p*<0.05, ***p*<0.01, ****p*<0.001).
