# Supplementary material for: Topical fluoride hesitancy and opposition are significantly and positively associated: A cross-sectional study
Source: PLoS One. 2025 Apr 30;20(4):e0322027. doi: 10.1371/journal.pone.0322027 (PMC12043125; doi:10.1371/journal.pone.0322027)
Supplement: S2 Table — (DOCX) [file pone.0322027.s002.docx]

**S2 Table.** Sociodemographic Characteristics of Surveyed Caregivers and Topical Fluoride Hesitancy Severity (N=1,042)

|  | **Severity of Topical Fluoride Hesitancy by Number of Domains, n (%)** | | | | | |  |
| --- | --- | --- | --- | --- | --- | --- | --- |
| **Sociodemographic Characteristic** | **0** | **1** | **2** | **3** | **4** | **5** | ***p*** |
| **Total n (%) for the domain** | 177 (17.6) | 442 (43.9) | 94 (9.3) | 68 (6.8) | 77 (7.7) | 148 (14.7) |  |
| **Child Gender** |  |  |  |  |  |  | .96 |
| Boy | 87 (49.7) | 213 (48.7) | 46 (49.5) | 34 (50.0) | 42 (54.5) | 70 (47.9) |  |
| Girl | 88 (50.3) | 224 (51.3) | 47 (50.5) | 34 (50.0) | 35 (45.5) | 76 (52.1) |  |
| **Child Age,** *mean±SD* | 7.7±4.5 | 8.1±4.3 | 7.7±4.0 | 8.5±5.1 | 7.1±4.5 | 6.8±4.1 | **.04*** |
| **Child Health Insurance Type** |  |  |  |  |  |  | **.01*** |
| Private | 82 (47.1) | 180 (41.2) | 34 (36.6) | 23 (34.3) | 19 (25.0) | 41 (28.3) |  |
| Medicaid/public | 89 (51.1) | 225 (51.5) | 52 (55.9) | 39 (58.2) | 52 (68.4) | 91 (62.8) |  |
| No insurance | 1 (0.6) | 9 (2.1) | 4 (4.3) | 1 (1.5) | 3 (3.9) | 4 (2.8) |  |
| Other | 2 (1.1) | 23 (5.3) | 3 (3.2) | 4 (6.0) | 2 (2.6) | 9 (6.2) |  |
| **Caregiver Gender** |  |  |  |  |  |  | .33 |
| Man | 30 (17.3) | 88 (20.7) | 19 (21.8) | 11 (16.9) | 14 (20.0) | 37 (27.2) |  |
| Woman | 141 (81.5) | 337 (79.1) | 68 (78.2) | 53 (81.5) | 55 (78.6) | 98 (72.1) |  |
| Non-binary or third gender | 2 (1.2) | 1 (0.2) | 0 (0.0) | 1 (1.5) | 1 (1.4) | 0 (0.0) |  |
| Prefer to self-identify as other | 0 (0.0) | 0 (0.0) | 0 (0.0) | 0 (0.0) | 0 (0.0) | 1 (0.7) |  |
| **Caregiver Age,** *mean±SD* | 42.9±8.0 | 41.9±8.1 | 41.8±7.8 | 42.0±10.5 | 41.1±8.4 | 42.2±8.9 | .48 |
| **Caregiver Race** |  |  |  |  |  |  | **<.001***** |
| White | 130 (76.9) | 240 (58.1) | 52 (61.2) | 26 (43.3) | 34 (50.7) | 56 (43.1) |  |
| Black | 4 (2.4) | 39 (9.4) | 3 (3.5) | 6 (10.0) | 11 (16.4) | 15 (11.5) |  |
| Asian | 17 (10.1) | 65 (15.7) | 13 (15.3) | 14 (23.3) | 13 (19.4) | 43 (33.1) |  |
| Multiple/Other | 18 (10.7) | 69 (16.7) | 17 (20.0) | 14 (23.3) | 9 (13.4) | 16 (12.3) |  |
| **Caregiver Ethnicity** |  |  |  |  |  |  | .75 |
| Hispanic | 21 (12.2) | 60 (14.3) | 8 (9.3) | 10 (16.4) | 11 (15.9) | 17 (12.4) |  |
| Non-Hispanic | 151 (87.8) | 360 (85.7) | 78 (90.7) | 51 (83.6) | 58 (84.1) | 120 (87.6) |  |
| **Caregiver Education** |  |  |  |  |  |  | **.046*** |
| Less than high school diploma | 1 (0.6) | 10 (2.3) | 2 (2.3) | 3 (4.7) | 4 (5.9) | 5 (3.7) |  |
| High school diploma or equivalent | 17 (9.9) | 53 (12.4) | 9 (10.3) | 6 (9.4) | 12 (17.6) | 25 (18.4) |  |
| Some college or 2-year college degree | 44 (25.6) | 111 (26.0) | 30 (34.5) | 21 (32.8) | 22 (32.4) | 48 (35.3) |  |
|  |  |  |  |  |  |  |  |

| **S2 Table (continued).** Sociodemographic Characteristics of Surveyed Caregivers and Topical Fluoride Hesitancy Severity (N=1,042) | | | | | | | |
| --- | --- | --- | --- | --- | --- | --- | --- |
| **Sociodemographic Characteristic** | **Severity of Topical Fluoride Hesitancy by Number of Domains, n (%)** | | | | | |  |
|  | **0** | **1** | **2** | **3** | **4** | **5** | ***p*** |
| 4-year college degree | 46 (26.7) | 110 (25.8) | 19 (21.8) | 18 (28.1) | 17 (25.0) | 28 (20.6) |  |
| More than 4-year college degree | 64 (37.2) | 143 (33.5) | 27 (31.0) | 16 (25.0) | 13 (19.1) | 30 (22.1) |  |
| **Parenting Style Scale,** *mean±SD* | 0.5±0.5 | 0.6±0.5 | 0.7±0.5 | 0.7±0.5 | 0.5±0.5 | 0.7±0.6 | **.006**** |
| **Caregiver Religiosity** |  |  |  |  |  |  | **.008**** |
| Very important | 46 (26.4) | 143 (33.3) | 30 (33.3) | 27 (42.9) | 28 (40.0) | 58 (41.4) |  |
| Somewhat important | 38 (21.8) | 102 (23.7) | 18 (20.0) | 21 (33.3) | 18 (25.7) | 43 (30.7) |  |
| Not too important | 34 (19.5) | 74 (17.2) | 18 (20.0) | 7 (11.1) | 10 (14.3) | 18 (12.9) |  |
| Not at all important | 56 (32.2) | 111 (25.8) | 24 (26.7) | 8 (12.7) | 14 (20.0) | 21 (15.0) |  |
| **Caregiver Political Ideology** |  |  |  |  |  |  | **<.001***** |
| Very conservative | 4 (2.3) | 7 (1.7) | 2 (2.4) | 0 (0.0) | 5 (7.4) | 14 (10.4) |  |
| Conservative | 14 (8.2) | 52 (12.3) | 5 (6.1) | 9 (15.3) | 7 (10.3) | 21 (15.7) |  |
| Moderate | 46 (26.9) | 180 (42.7) | 34 (41.5) | 27 (45.8) | 29 (42.6) | 57 (42.5) |  |
| Liberal | 57 (33.3) | 116 (27.5) | 28 (34.1) | 12 (20.3) | 17 (25.0) | 23 (17.2) |  |
| Very liberal | 50 (29.2) | 67 (15.9) | 13 (15.9) | 11 (18.6) | 10 (14.7) | 19 (14.2) |  |
| **Annual Household Income** |  |  |  |  |  |  | **.02*** |
| <$15,000 | 8 (4.7) | 26 (6.2) | 6 (7.2) | 5 (8.3) | 7 (10.3) | 10 (7.8) |  |
| $15,000 to <$25,000 | 11 (6.5) | 35 (8.4) | 10 (12.0) | 7 (11.7) | 10 (14.7) | 14 (10.9) |  |
| $25,000 to <$50,000 | 32 (18.9) | 80 (19.2) | 13 (15.7) | 14 (23.3) | 21 (30.9) | 39 (30.2) |  |
| $50,000 to <$75,000 | 24 (14.2) | 76 (18.2) | 17 (20.5) | 14 (23.3) | 10 (14.7) | 22 (17.1) |  |
| $75,000 to <$100,000 | 16 (9.5) | 50 (12.0) | 14 (16.9) | 5 (8.3) | 7 (10.3) | 11 (8.5) |  |
| $100,000 to <$150,000 | 31 (18.3) | 72 (17.3) | 12 (14.5) | 8 (13.3) | 7 (10.3) | 20 (15.5) |  |
| ≥$150,000 | 47 (27.8) | 78 (18.7) | 11 (13.3) | 7 (11.7) | 6 (8.8) | 13 (10.1) |  |

*Note*: Boldface indicates statistical significance (**p*<0.05, ***p*<0.01, ****p*<0.001).
