# Supplementary material for: Topical fluoride hesitancy and opposition are significantly and positively associated: A cross-sectional study
Source: PLoS One. 2025 Apr 30;20(4):e0322027. doi: 10.1371/journal.pone.0322027 (PMC12043125; doi:10.1371/journal.pone.0322027)
Supplement: Attachment — Submitted filename: Response to Reviewers.docx [file pone.0322027.s005.docx]

**PONE-D-24-50799
Topical fluoride hesitancy and opposition are significantly and positively associated**

**Response to Reviewers**

Thank you for reviewing our manuscript and providing us the opportunity to revise. Please see below for a point-by-point response to the reviewers’ comments. All page numbers refer to the revised manuscript with track changes file.

Reviewer #1: The manuscript on topical fluoride hesitancy and opposition presents a well-structured study focused on understanding the relationship between hesitancy and opposition to fluoride among caregivers. Here’s a review addressing its strengths and areas for improvement.

Strengths

Clear Research Focus: The study’s objective—to explore the potential link between topical fluoride hesitancy and opposition—is clearly defined and addresses a relevant topic in preventive dental care, especially given the rise of vaccine and medical treatment hesitancy in recent years.

Comprehensive Survey Design: The use of an 85-item survey with a specialized Fluoride Hesitancy Identification Tool (FHIT) allowed the authors to capture nuanced data across five domains (necessity, chemicals, harm, uncertainty, distrust), leading to detailed insights. This breadth supports the robustness of the findings.

Large Sample Size and Diverse Population: With over a thousand caregivers included, the sample size is considerable and provides a good demographic mix. The data on caregivers’ age, gender, and race allow for generalizable results and may contribute to understanding diverse perspectives on fluoride.

Statistical Rigor: The study applies confounder-adjusted logistic regression models to assess the association between fluoride hesitancy and opposition. This strengthens the validity of the findings, as the analysis accounts for potential confounders, making the relationship between hesitancy and opposition more credible.

Practical Implications: The conclusion that hesitancy is positively associated with opposition, although distinct, can inform public health strategies. Understanding these nuances could help in designing interventions to mitigate fluoride hesitancy before it leads to opposition, potentially impacting oral health at a population level**.**
**RESPONSE: Thank you for your careful review. Below we provide responses to each suggestion.**

Areas for Improvement

**Reviewer 1 Comment 1:** 
Clarification of Domain-Specific Findings: While domain-specific hesitancy percentages are reported, the discussion could benefit from deeper insights into how specific domains (e.g., distrust or perceived harm) contribute more significantly to opposition. This could reveal which beliefs are most critical to address in order to prevent opposition.
**RESPONSE: Thank you for the careful review. Further insight has been included in the Discussion section to discuss the domains that significantly contributed to opposition, which were chemicals and necessity (Page 18, Lines 310-312).**

**Reviewer 1 Comment 2**:
Potential for Causation or Progression Analysis: The manuscript suggests that hesitancy may be a precursor to opposition, yet no longitudinal analysis was conducted to assess if individuals progress from hesitancy to opposition over time. Future research could strengthen this hypothesis by employing a longitudinal design to explore whether hesitancy in specific domains consistently leads to opposition.
**RESPONSE: We agree with this comment, with acknowledgment of this need in the Discussion section (Page 18 Lines 294-295, 303-305; Page 22 Lines 402-404).**

**Reviewer 1 Comment 3**:
Sample Representativeness: While the study’s sample includes caregivers of varied demographics, it may still benefit from a discussion on the potential influence of regional attitudes or socio-economic factors on fluoride hesitancy and opposition. Including more details about geographic, economic, or educational backgrounds could help clarify any limitations in generalizing the findings.
**RESPONSE: Thank you for this feedback. Additional points focusing on geographic and political leanings to clarify limitations, including comparisons to US Census data, were included in the Discussion section and Limitations section (Page 17 Lines 266-277; Page 21 Lines 388-390; Page 22 Lines 391-401).**

**Reviewer 1 Comment 4**:
Discussion of Intervention Strategies: Although the authors call for further research to understand the link between hesitancy and opposition, the study would benefit from an expanded discussion on actionable strategies based on the findings. For instance, suggesting specific public health or educational interventions targeting the high hesitancy levels observed in the “necessity” domain could add practical value.
**RESPONSE: Thank you for this feedback. The discussion on actionable strategies was expanded as suggested in the Discussion section (Page 19 Lines 318-324; Page 20 Lines 346-362; Page 21 Lines 373-374).**

**Reviewer 1 Comment 5**:
Addressing Potential Biases: Given that a large percentage of respondents were female and white, any inherent biases from these demographics might influence perceptions of fluoride. It could be beneficial to discuss how such demographics may shape views on topical fluoride and whether the findings might differ in more balanced or varied demographic groups.
**RESPONSE: Thank you for the feedback. Additional discussion of these demographics has been included in the Discussion section (Page 17 Lines 273-277).**

**Reviewer 2 Comment 1**:
While I appreciate the research was conducted and paper written prior to the recent pending change in leadership in the US, I think it is worth including the potential impact of the findings in the context of this change, especially in the light of the widely reported possibility of the cessation of water fluoridation across the US. While this may not happen there are significant fears for (dental) public health in the US. This pending change can be weaved into the Introduction of the paper but more critically into the Discussion. The Discussion has the potential to be more insightful and provide evidence-based strategies to address fluoride hesitancy and opposition. The Discussion essentially compares findings where possible and attempts to explain the findings. However the reader the left with little more. The need for a longitudinal study is mentioned a few times, which is fine, but the focus should be the current findings and the current and near future context of these findings.
**RESPONSE: We agree with this comment. This was addressed by addressing the reported possible changes to water fluoridation in the Introduction (Page 3 Lines 62-69) and an emphasis in the Discussion on strategies based on current findings to address fluoride hesitancy (Page 20 Lines 346-362; Page 21 Lines 373-374).**

**Reviewer 2 Comment 2**:
The conclusion includes findings from other studies. The conclusion should be related to the current study (findings) and some information here could be moved into the Discussion.
**RESPONSE: Thank you for the feedback. Some information was moved to the Discussion (Page 20 Lines 346-354) and the conclusion was edited to exclusively discuss the current study and findings (Page 22 Lines 410-412).**

**Reviewer 2 Comment 3**:
For non-US readers it may be useful to provide more information about the study setting (how do people of the State vote?) In the recent election the President-Elect received about 39% of the vote. I would guess hesitancy and in particular opposition would be much higher in the States which overwhelmingly voted for the President-Elect.
What does mean for the country? People I think will now be more embolden to express this hesitancy and opposition publicly and to their health care providers.
**RESPONSE: We did not elaborate on the U.S. election process to stay focused on the main topic, but we agree this is an important element to address. Effects of recent political changes and the impact of conservatism on the study results were expanded on in the Introduction and Discussion sections (Page 3 Lines 62-69; Page 17 Lines 266-273).**

**Reviewer 2 Comment 4**:
The generalizability of the findings is identified as a limitation, however how representative is the sample of the US population, for example in terms of race, ethnicity, education, religiosity, ideology and annual income?
**RESPONSE: We agree with this comment that how representative the same is of the US population must be addressed. This is expanded on with US Census data in the Limitations section (Page 22 Lines 393-401).**

**Reviewer 2 Comment 5**:
In the tables would it make sense to report row rather than column percentages?
**RESPONSE: Tables 1 and 2 report column percentages as intended to allow readers to easily see whether the bivariate relationships are statistically significant. Therefore, no changes were made to Tables in the revised submission.**

**Reviewer 3 Comment 1**:
The manuscript should conform to the Strengthening the Reporting of Observational Studies in Epidemiology (STROBE) guidelines. Please use the STROBE checklist for reference. [STROBE website]([https://www.strobe-statement.org/](https://urldefense.com/v3/__https:/www.strobe-statement.org/__;!!K-Hz7m0Vt54!md86XDDVmDw5ShhvizbjN5jwuDk7EhWBloqxdGSnOaVIO6HSDbqsqmZx6_RB0wwwEEOmae1c5oa8$)).
**RESPONSE: Thank you for the careful review. We revised the manuscript throughout to conform to the STROBE guidelines using the checklist. The checklist has been included with the revision.**

**Reviewer 3 Comment 2:**
The study primarily uses a sample from a single urban area in Washington state. However, the Introduction (Page 3, Line 49-51)(Page 4, Line 75-78), Discussion (Page 18, Lines 291-294)(Page 18, Line 296-298), and Conclusion (Page 21, Lines 361-362) focus on caregivers of children at high risk of dental caries. It is necessary to clarify how many participants were at high risk or whether all individuals in the sample are considered to require topical fluoride applications for the generalizability of the study.
**RESPONSE: We did not assess caries risk for study participants. However, we did include questions on whether the child has ever had a cavity and likelihood of getting a cavity in the future. We clarified how many participants were high-risk and further detail in the Discussion section (Page 19, Lines 327-335).**

**Reviewer 3 Comment 3:**
3. Please provide the full name of REDCap and include its reference (Page 5, Line 96).
**RESPONSE: The full name and reference were provided as suggested (Page 5 Line 103; Page 26 Line 490).**

**Reviewer 3 Comment 4:**
4. Please specify where Mplus was obtained (Page 9, Line 140).
**RESPONSE: An in-text citation and reference were added to provide readers with information on Mplus and the ability to buy Mplus (Page 9 Line 150; Page 27 Lines 496-497).**

**Reviewer 3 Comment 5:**
5. Dental caries risk factors should encompass more than just fluoride exposure and sugar intake. Please consider expanding on other relevant factors (Page 18, Lines 297-298).
**RESPONSE: This is a valid concern. We expanded on the consideration of other relevant factors, while emphasizing these two substantial behavioral risk factors that are relevant to the current study (added sugars and fluoride) (Page 19 Lines 335-338).**
