# Supplementary material for: Topical fluoride hesitancy and opposition are significantly and positively associated: A cross-sectional study
Source: PLoS One. 2025 Apr 30;20(4):e0322027. doi: 10.1371/journal.pone.0322027 (PMC12043125; doi:10.1371/journal.pone.0322027)
Supplement: Attachment — Submitted filename: Response_to_Reviewers_auresp_2.docx [file pone.0322027.s006.docx]

**PONE-D-24-50799R1
Topical fluoride hesitancy and opposition are significantly and positively associated: A cross-sectional study**

**Response to Reviewers**

Thank you for reviewing our manuscript and providing us the opportunity to revise. Please see below for a point-by-point response to the reviewers’ comments. All page numbers refer to the revised manuscript with track changes file.

Reviewer #1: While the manuscript aims to address an important topic—the relationship between topical fluoride hesitancy and opposition—it has several significant shortcomings that hinder its overall quality and contribution to the field.

Lack of Novelty:
The manuscript does not provide sufficient novelty or groundbreaking insights. The association between hesitancy and opposition is a logical assumption that lacks a compelling rationale or unique contribution. The findings largely reiterate well-established concepts without exploring deeper mechanisms or implications.

**RESPONSE: Thank you for your careful review. While the association is a logical assumption, we highlight in the Introduction (Page 4 Lines 82-87) the necessity of empirical research on the subject.**

Methodological Weaknesses:
Survey Design: The use of an 85-item survey may have introduced response fatigue among participants, potentially compromising the reliability of the responses. The manuscript does not address steps taken to mitigate this risk.

**RESPONSE: Thank you for this feedback. The introduction of response fatigue is a valid concern. This limitation was addressed by pre-testing the survey with caregivers, ensuring that all items were written at the fourth-grade level. Most caregivers completed the questionnaire while their children were getting their teeth cleaned.**

Validation of FHIT Tool: The manuscript does not provide adequate evidence supporting the validity and reliability of the Fluoride Hesitancy Identification Tool (FHIT) in this specific population, raising concerns about the accuracy of the hesitancy scores.

**RESPONSE: Thank you for your careful review. The validity and reliability of the FHIT are supported by three studies cited in the Measures section (Page 5 Line 113).**

Sample Representation: Although the study includes 1,042 caregivers, the demographic breakdown (e.g., predominantly female and white participants) limits the generalizability of the findings to more diverse populations.

**RESPONSE: We agree with this comment, with acknowledgment of the limited generalizability of the study and comparisons to the most recent U.S. Census data in the Limitations section (Page 22 Lines 398-405).**

Insufficient Context and Justification:
The manuscript fails to sufficiently contextualize why topical fluoride hesitancy is a critical issue requiring such detailed investigation. A stronger argument connecting this hesitancy to significant public health outcomes would enhance its relevance.
**RESPONSE: Thank you for the careful review. Further contextualization and a reference have been included in the Introduction section to indicate the importance of this issue (Page 3, Lines 52-53; Page 24 Lines 452-454; Page 25 Lines 455-456).**

The discussion lacks depth in exploring underlying factors driving hesitancy and opposition. Socioeconomic, cultural, or psychological determinants are notably absent.
**RESPONSE: Thank you for the careful review. We agree that these determinants are an important consideration. To address this, we have expanded upon our discussion of future studies on caregivers from broader socioeconomic backgrounds to include all mentioned determinants in the Discussion section (Page 17 Lines 281-282; Page 18 Lines 285-286).**

Superficial Statistical Analysis:
While the study uses logistic regression models, the manuscript does not delve into the potential influence of confounding variables beyond superficial adjustments. It would be more informative to explore interaction effects or subgroup analyses to provide nuanced insights.
**RESPONSE: We agree these are potentially important issues but they are beyond the scope of the current study. We hope to address these questions in future studies.**

Overgeneralized Conclusions:
The conclusion that "topical fluoride hesitancy and opposition are positively associated but not identical" is vague and does not meaningfully contribute to actionable strategies. The manuscript offers little practical guidance for addressing hesitancy or preventing opposition.

**RESPONSE: Thank you for the careful review. The first statement in the conclusion seeks to identify the main findings of the analysis, which are that a positive association is found between hesitancy and opposition, and the difference in prevalence of the two variables point to a distinction between the two, as expanded in the Discussion section (Page 17 Lines 288-291; Page 18 Lines 292-301). Practical guidance was provided in the Discussion section as well to address hesitancy and opposition (Page 19 Lines 320-328, 344-346, 359-367, 369-381).**

Writing and Presentation Issues:
The text is verbose and repetitive, which detracts from readability.
There are inconsistencies in terminology, such as fluctuating references to "general hesitancy" and "domain-specific hesitancy," which could confuse readers.
**RESPONSE: Thank you for the feedback. “General hesitancy” and “domain-specific hesitancy” are separate terms defined in the Measures section (Page 6 Lines 123-128).**

Recommendations for Improvement
Provide a more compelling justification for the study, explicitly linking hesitancy and opposition to broader public health outcomes.
Include a detailed validation process for the FHIT tool or use a more established instrument.
Address the limitations of the sample's demographic homogeneity and consider analyzing underrepresented groups separately.
Strengthen statistical analyses by incorporating more sophisticated methods, such as mediation or path analysis, to elucidate the mechanisms linking hesitancy and opposition.
Revise the discussion and conclusion to provide specific, actionable recommendations for public health interventions.
Edit the manuscript for conciseness and consistency, ensuring clear and precise language.
In its current form, the manuscript lacks sufficient originality, methodological rigor, and practical value to justify publication. Significant revisions are required to enhance its scientific and public health impact.

**Thank you for the careful review. The recommendations for improvement are individually addressed as described above.**

Reviewer #2: (No Response)

**Reviewer 3 Comment 1:**
Thank you for including the STROBE checklist. You can state that the manuscript conforms to the STROBE guidelines in the Materials and Methods section.

**RESPONSE: Thank you for the careful review. A statement on conforming to the STROBE guidelines in the Materials and Methods section, as well as a citation have been added (Page 5 Line 104; Page 27 Lines 500-501).**


**Reviewer 3 Comment 2:**
Thank you for clarifying the estimation of high-risk individuals, the concerns of low-risk individuals, and the justification for addressing topical fluoride hesitancy in the study population. The indicated line numbers are incorrect.

**RESPONSE: Thank you for the feedback. The line numbers have been corrected accordingly (Page 19 Lines 332-340).**


**Reviewer 3 Comment 3:**
Thank you for providing the full name and reference of REDCap. The indicated line numbers are incorrect.

**Thank you for the feedback. The line numbers have been corrected accordingly (Page 5 Lines 107-108; Page 27 Line 502).**


**Reviewer 3 Comment 4:**
Thank you for providing the information on Mplus. The indicated line numbers are incorrect.
**Thank you for the feedback. The line numbers have been corrected accordingly (Page 9 Line 154; Page 27 Lines 508-509).**

**Reviewer 3 Comment 5:**
Thank you for expanding on other relevant factors of dental caries risk. Please include cariogenic bacteria, which is fundamental to the Keyes triad. The indicated line numbers are incorrect.

**Thank you for the feedback. Discussion of cariogenic bacteria was added and the line numbers have been corrected accordingly (Page 19 Lines 340-343; Page 20 Line 344).**
